# C-reactive Protein Elevation Is Associated With QTc Interval Prolongation in Patients Hospitalized With COVID-19

**DOI:** 10.3389/fcvm.2022.866146

**Published:** 2022-06-23

**Authors:** Nino Isakadze, Marc C. Engels, Dominik Beer, Rebecca McClellan, Lisa R. Yanek, Bahareh Mondaloo, Allison G. Hays, Thomas S. Metkus, Hugh Calkins, Andreas S. Barth

**Affiliations:** ^1^Division of Cardiology, Department of Medicine, Johns Hopkins University School of Medicine, Baltimore, MD, United States; ^2^Department of Medicine, Johns Hopkins University School of Medicine, Baltimore, MD, United States

**Keywords:** C-reactive protein, QTc interval, COVID-19, inflammatory markers, arrhythmia

## Abstract

**Background:**

The relationship between inflammation and corrected QT (QTc) interval prolongation is currently not well defined in patients with COVID-19.

**Objective:**

This study aimed to assess the effect of marked interval changes in the inflammatory marker C-reactive protein (CRP) on QTc interval in patients hospitalized with COVID-19.

**Methods:**

In this retrospective cohort study of hospitalized adult patients admitted with COVID-19 infection, we identified 85 patients who had markedly elevated CRP levels and serial measurements of an ECG and CRP during the same admission. We compared mean QTc interval duration, and other clinical and ECG characteristics between times when CRP values were high and low. We performed mixed-effects linear regression analysis to identify associations between CRP levels and QTc interval in univariable and adjusted models.

**Results:**

Mean age was 58 ± 16 years, of which 39% were women, 41% were Black, and 25% were White. On average, the QTc interval calculated *via* the Bazett formula was 15 ms higher when the CRP values were “high” vs. “low” [447 ms (IQR 427–472 ms) and 432 ms (IQR 412–452 ms), respectively]. A 100 mg/L increase in CRP was associated with a 1.5 ms increase in QTc interval [β coefficient 0.15, 95% CI (0.06–0.24). In a fully adjusted model for sociodemographic, ECG, and clinical factors, the association remained significant (β coefficient 0.14, 95% CI 0.05–0.23).

**Conclusion:**

An interval QTc interval prolongation is observed with a marked elevation in CRP levels in patients with COVID-19.

## Introduction

COVID-19, an inflammatory disease state, is associated with significantly elevated systemic inflammatory markers (1–3). COVID-19 has also been associated with an increased risk of cardiac arrhythmias, including life-threatening arrhythmias associated with corrected QT (QTc) interval prolongation (4–6). While the etiology of arrhythmias in this cohort of patients is thought to be multifactorial, including direct myocyte injury, multiorgan dysfunction, and medications that prolonged the QT interval, the role of inflammation on cardiac repolarization has not been robustly explored (7, 8).

Prolongation of the QTc interval is associated with an increased risk of ventricular arrhythmias and sudden cardiac death (9). Many reversible factors have been identified that lead to acquired QT prolongation, including QTc-prolonging medications and metabolic/electrolyte changes, among others (10, 11). Acute and chronic inflammatory states are emerging as risk factors associated with acquired QTc prolongation and an increased risk of torsades de pointes and mortality (12–19). Understanding whether a marked elevation in the inflammatory marker C- reactive protein (CRP) is associated with changes in the QTc interval among hospitalized patients with COVID-19 will help guide clinical decision making when considering initiation of QTc prolonging medications and managing other factors that lead to acquired QTc prolongation. Furthermore, a better understanding of the relationship between the inflammatory marker CRP and the QTc interval among individuals hospitalized for COVID-19 will add to the growing body of the literature on the role of inflammation in acquired prolongation of QTc interval.

## Methods

We conducted a retrospective cohort study of 85 adult individuals who were admitted for treatment of acute COVID-19 infection to one of five hospitals within the Johns Hopkins Health System in the Baltimore/Washington, DC metropolitan area between 1 March 2020 and 20 September 2020. Data were obtained from the COVID-19 Precision Medicine Analytics Platform Registry (JH-CROWN). The workflow and inclusion criteria are presented in Supplementary eFigure 2. We included individuals who had serial measurements of ECG and CRP on two sequential dates during the same hospitalization, one defined as “high” when CRP was five or more times above the upper limit of normal and the other defined as “low” when CRP level was <50% of the peak value. We focused our analysis on individuals with such variation in CRP levels, given that the effect of marked CRP changes (e.g., >75%) was shown to affect cardiac repolarization in a prior study (20). ECGs were obtained within 24 h of the CRP measurements. Patients with a QRS duration of ≥120 ms or atrial fibrillation were excluded as these conditions can affect accurate QTc assessment (21, 22). The study was approved by the Johns Hopkins Institutional Review Board (IRB00248707). Given the retrospective chart review design, informed consent was waived.

Baseline and interval characteristics of individual patients, including demographics and ECG data, were extracted from the JH-CROWN registry. Automatic data extraction was supplemented by manual electronic medical record review to obtain ECG data (heart rate, PR interval, QRS interval, and QT/QTc interval), electrolyte levels, creatinine level, medication list, and clinical characteristics to calculate the COVID-19 WHO disease severity score from two separate dates (high CRP and low CRP dates) for each individual. Automated QT measurements were manually verified by two independent reviewers, including a cardiac electrophysiologist. The number of QTc prolonging medications (identified based on www.crediblemeds.org) was determined from the list of medications administered during high CRP and low CRP dates for each patient. The QTc interval was calculated primarily using the Bazett formula, (23) however, given the poor performance of the Bazett's formula at faster heart rates, the Fridericia (24), Hodges (25), and Framingham (26) formulae were also calculated.

The severity of COVID-19 was determined by calculation of the WHO scale, (27, 28) which is an eight-point ordinal scale. Scores of 1 and 2 correspond to an ambulatory clinical state with 1 being asymptomatic and 2 being mildly limited in activity. Scores 3–7 correspond to a hospitalized state with 3 signifying a patient on room air, 4—requiring supplemental oxygen via nasal cannula, 5—requiring high flow nasal cannula or noninvasive positive pressure ventilation, 6—requiring intubation and mechanical ventilation, and 7—requiring intubation and mechanical ventilation as well as the presence of signs of organ failure. A score of 8 corresponds to death.

Statistical analysis was performed using Stata 17 software (StataCorp, College Station, TX, United States). Continuous variables are presented as mean ± standard deviation if variables were normally distributed and medians and interquartile ranges when variables deviated from the normal distribution. Normality was assessed by plotting quantiles of variables against quantiles of normal distribution (Supplementary eFigure 1). To compare characteristics between the two-time points, we used a paired t-test if variables were continuous and normally distributed and the Wilcoxon signed-rank test when continuous variables deviated from the normal distribution. We used the McNemar-Bowker test for comparing multiple correlated proportions for categorical variables containing more than two categories. A two-tailed p-Value cutoff of <0.05 was used to determine statistical significance for all analyses.

We performed mixed-effects linear regression in crude and adjusted models to estimate the effect of CRP levels on QTc interval. Our two levels or groups were sequential time points of ECG and CRP measures in individual patients. In addition to demographic variables, we included clinical and ECG characteristics that have been shown to be associated with exposure or outcome in our analysis (disease severity and heart rate, Table 1 and QTc prolonging medications, Supplementary eTable 1) as fixed effects in the adjusted analysis with random intercept and slope. Further variable inclusion that has been previously shown to be associated with both exposure and outcome or outcome alone, such as PR interval, QRS duration, potassium level, or creatinine level was limited due to the small sample size. We first performed an unadjusted analysis and then adjusted for demographic variables (age, sex, and race) in Model 1. In Model 2, we adjusted for demographic variables (age and sex), ECG characteristics [(heart rate, disease severity (WHO score)], and number of QTc prolonging medications used.

**Table 1:** Baseline demographic and clinical characteristics by high and low CRP days, *n* = 85.

	**High CRP median (IQR), mg/L 222 (147–561)**	**Low CRP Median (IQR), mg/L 25 (11–65)**	*P*-Value*
**Demographic characteristics**	58 (16)		
Age (years)—mean (SD)			
Female sex—no. (%)	33 (39%)		
**Race—no. (%)**			
White	21 (25%)		
Black	35 (41%)		
Other	29 (34%)		
**Clinical characteristics**			
BMI (kg/m^2^)—median (IQR)	29 (25–32)		
WHO score*–number of patients (%)			
3—no supplemental oxygen requirement	10 (12)	22 (26)	
4—requiring supplemental oxygen *via* nasal cannula	27 (32)	25 (30)	0.03
5—requiring high flow nasal cannula or noninvasive positive pressure ventilation	5 (6)	4 (5)	
6—requiring intubation and mechanical ventilation	13 (16)	13 (16)	
7—requiring intubation and mechanical ventilation plus signs of organ insufficiency	29 (35)	20 (24)	
**ECG characteristics**			
HR (bpm)—median (IQR)	87 (77–98)	81 (68–96)	0.06
PR Interval (ms)—median (IQR)	154 (138–171)	160 (140–175)	0.14
QRS duration (ms)—mean (SD)	87.67 (10)	88.76 (11)	0.42
QT interval** (ms)—median (IQR)	447 (427–472)	432 (412–452)	0.0001
**Number of QTc prolonging medications (%)**			
0	48 (56)	47 (55)	
1	26 (31)	23 (27)	0.26
≥2	11 (13)	15 (18)	
**Laboratory characteristics**			
Creatinine (mg/dl)—median (IQR)	1 (0.7–1.5)	0.8 (0.7–1.5)	0.04
Potassium (mmol/L)—median (IQR)	4.2 (3.8–4.5)	4.2 (3.9–4.4)	0.3
Magnesium*** (mg/dl ) median IQR)	2.2 (1.9–2.4)	2.2 (2–2.4)	0.4

*Bpm, beats per minute; BMI, body mass index; CRP, C reactive protein; HR, heart rate; IQR, interquartile range; QTc, corrected QT interval; SD, standard deviation; WHO, World Health Organization*.

^*^P-Values are based on paired t-test for normally distributed continuous variables. Wilcoxon signed rank test was used when continuous variables were not distributed normally and Bowker test for multiple correlated proportions for categorical variables containing more than two categories.

^**^Measured by Bazett formula.

^***^Magnesium variable was missing in 38% of patient population.

## Results

**Figure 1:**
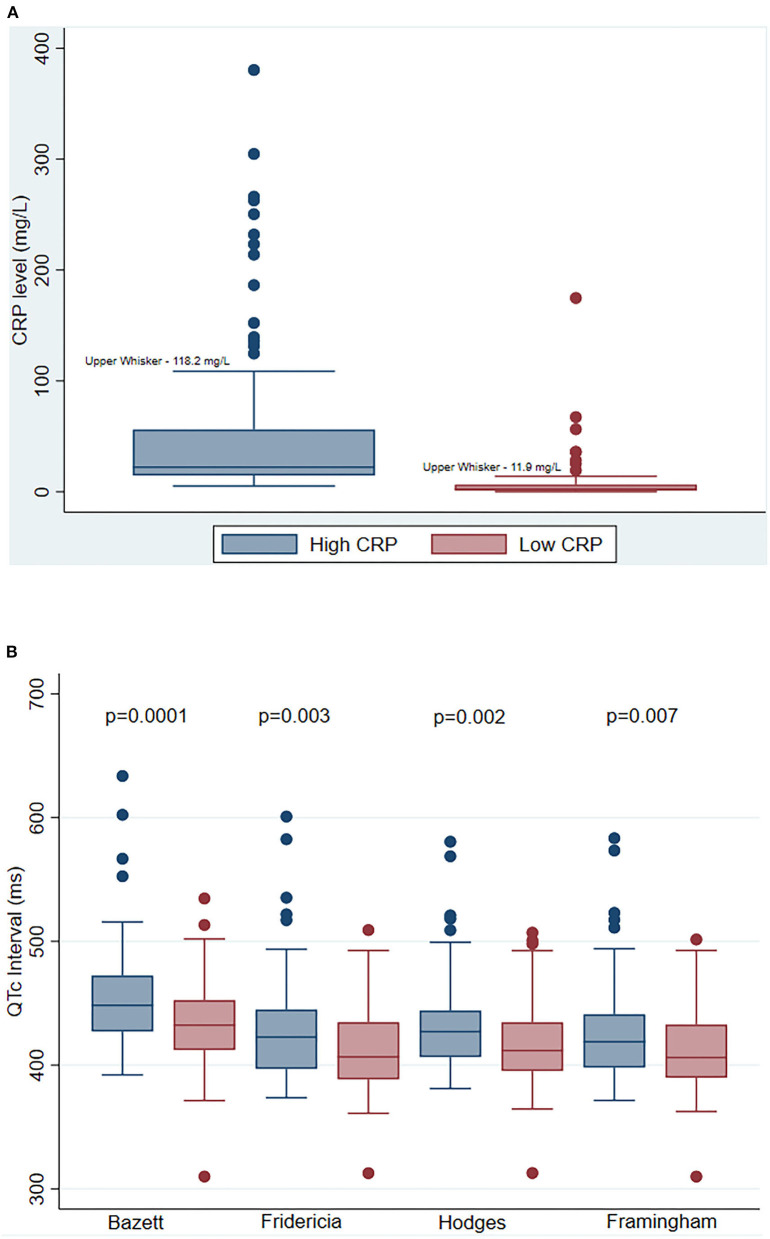
Serial change in C-reactive protein (CRP) and corrected QT (QTc) interval. **(A)** Comparison of median CRP levels during high and low CRP values. Each bar represents the median CRP level (+/– IQR) during high CRP values (blue), and during low CRP values (red). The P-Value indicates a statistical comparison between high CRP (blue) and low CRP (red) values. **(B)** Comparison of QTc interval measured by various correction methods at high and low CRP values. Each bar represents the median QTc level (+/– IQR) during high CRP values (blue), and during low CRP values (red) measured by Bazett, Fridericia, Hodges, and Framingham methods, respectively. The P-Values indicate statistical comparisons of QTc interval between high CRP (blue) and low CRP (red) values. Abbreviations: CRP, C-reactive protein; QTc, corrected QT interval.

**Figure 2:**
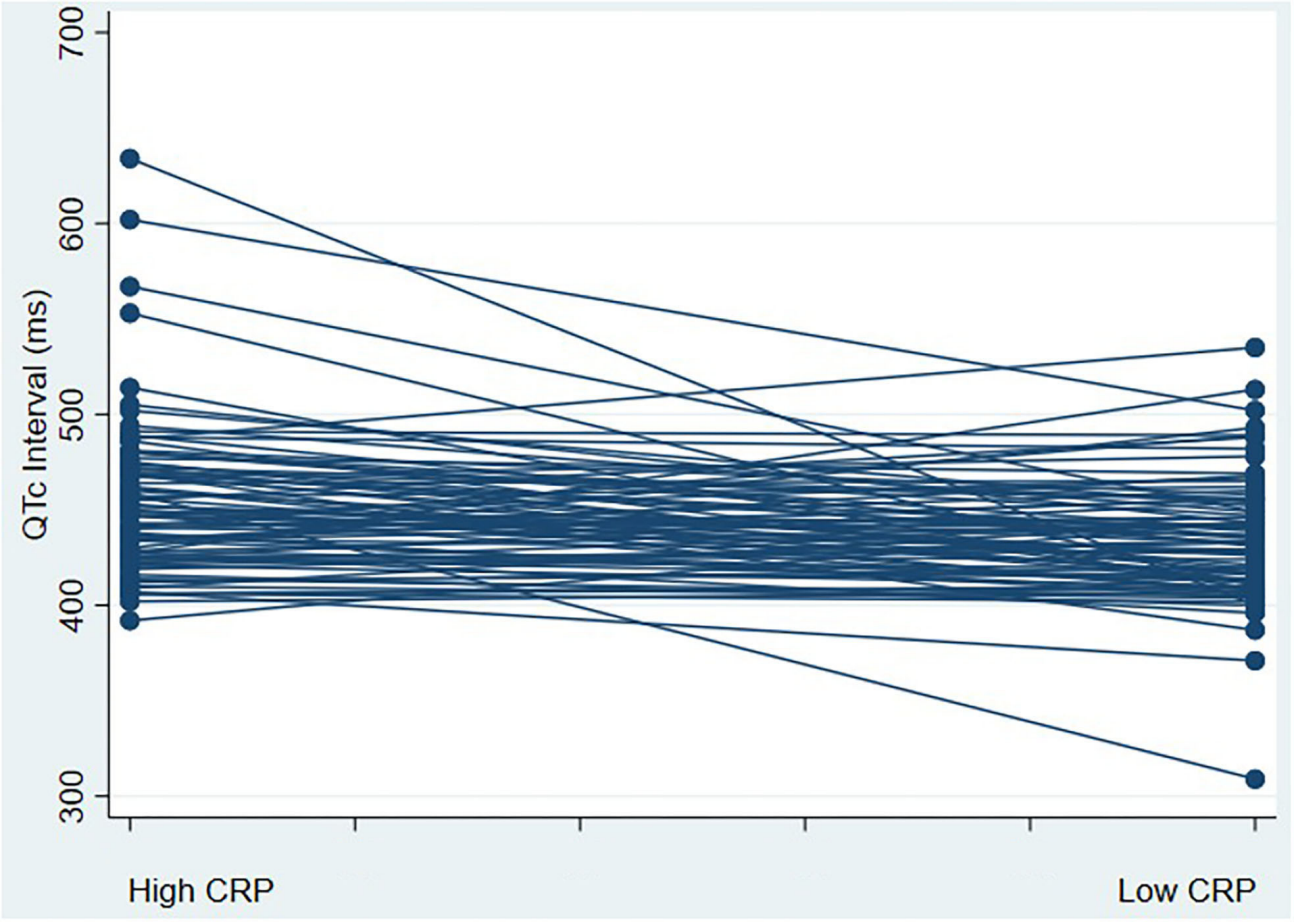
corrected QT **(QTc)** interval change with a marked change in CRP values. This figure represents a spaghetti plot of QTc interval values comparing QTc intervals during high and low CRP states; 27 participants had an increase in QTc interval with a decrease in CRP levels (discordant), whereas 58 participants had a decrease in QTc interval with a reduction in CRP levels (concordant changes). Abbreviations: CRP, C-reactive protein; QTc, corrected QT interval.

There were 85 patients hospitalized for COVID-19 who met the study eligibility criteria. Table 1 shows the baseline demographic and clinical characteristics of the study cohort. The mean age was 58 ± 16 years; 39% were women; 41% were Black, 25% White, and 34 % other. Median BMI was 29 (IQR 25–32) (Table 1). The median CRP values were 22.2 mg/L and 2.5 mg/L during high and low CRP days, respectively (Figure 1A). The mean difference between high and low CRP values was 11 days. QTc interval calculated via the Bazett formula was on average 15 ms higher [447 ms (IQR 427–472 ms) vs. 432 ms (IQR 412–452 ms) when the CRP values were high vs. low, respectively. The QTc interval was higher when CRP values were high vs. low, irrespective of which QT correction formula was used (Bazett, Fridericia, Hodges, or Framingham) (Figure 1B). Concordant changes between CRP levels and QTc were found in 58 patients, while 27 participants showed either no change or had an increase in QTc interval with a decrease in CRP levels (discordant changes; Figure 2). Moderate QTc prolongation (QTc >470 ms in men and >480 ms in women) (9) was observed in 10 men and nine women during high CRP and five men and four women during low CRP values. Furthermore, marked QTc prolongation >500 ms was observed among two men and five women during high CRP values and one man and two women during low CRP values. Extreme QTc prolongation (QTc values >550–600 ms) was observed among one man and three women with high CRP levels and no patients with low CRP values.

No statistically significant differences were found for ECG characteristics including heart rate, PR interval or QRS duration, and potassium levels when CRP values were high vs. low (Table 1). COVID-19 severity was worse (defined by the WHO scale), and creatinine levels were higher when CRP levels were high vs. low (Table 1). QTc interval in women was on average 15 ms (95% CI 3.2–27 ms) higher than that in men. One vs. no QTc prolonging medication use was associated with QTc prolongation, however, two vs. none was not, which was likely due to the very small sample size [95% CI coefficient 16.34 (95% CI 3.38–29.3), and coefficient 8.31 (−7.97 to 24.6), respectively]. Examples of commonly used QT-prolonging medications included azithromycin, quetiapine, and haloperidol. Other demographic, ECG, and clinical variables examined failed to reach significance in our analysis (Supplementary eTable 1).

Table 2 shows crude and adjusted mixed-effects linear regression of QTc interval on CRP level. In an unadjusted analysis, a 100 mg/L increase in CRP was associated with a 1.5 ms increase in QTc interval (β coefficient 0.15, 95% CI 0.06–0.24). After adjusting for sex and other sociodemographic variables (age and race) the association remained unchanged (β coefficient 0.15, 95% CI 0.06–0.23). When adjusted for ECG characteristics (heart rate), disease severity (WHO score), and number of QTc prolonging medications in addition to demographic variables, the association remained statistically significant with a slight attenuation of estimate (β coefficient 0.14, 95% CI 0.05–0.23).

**Table 2:** Crude and adjusted linear mixed effects regression of QTc interval on CRP level,demographics, ECG and clinical characteristics.

	**Linear mixed effects regression of QTc interval on CRP level**		
	**Crude**	**Adjusted** **(Model 1)**	**Adjusted** **(Model 2)**
	**β coefficient (95% CI)**	**β coefficient (95% CI)**	**β coefficient (95% CI)**
CRP	0.15 (0.06–0.24)	0.15 (0.06–0.23)	0.14 (0.05–0.23)

*CRP, C-reactive protein; ECG, electrocardiogram; QTc, corrected QT interval*.

*Model 1: adjusted for age, sex, race. Model 2: adjusted for age, sex, race, WHO score, heart rate, and number of QTc prolonging medications*.

## Discussion

In this retrospective cohort study of a racially diverse patient population hospitalized for COVID-19 who had marked elevations and interval changes in CRP levels, we found that an elevation of CRP levels was associated with a prolongation of the QTc interval. The major strength of our design is (1) that a racially diverse study population was examined and (2) that study participants serve as their own controls minimizing the risk of bias due to known and unknown confounders, while differences in disease severity and clinical factors at different time points were adjusted for.

In a small cohort of individuals with non-COVID-19 acute infections (mostly pneumonia), Lazzerini et al. found a significant correlation between QTc interval and inflammatory markers in an unadjusted analysis. After a marked reduction in CRP levels, the QTc interval duration decreased, and the degree of CRP reduction correlated with the decrease in QTc interval (20). The effect of increased systemic inflammation on QT prolongation and electrical instability is not only limited to acute infection. A prolonged QTc interval has been shown to be associated with chronically elevated inflammatory markers among individuals with rheumatoid arthritis and other connective tissue diseases (12, 29–31) who also have an increased risk of sudden cardiac death (32). Furthermore, the average QTc interval is longer among individuals with inflammatory bowel diseases and Human Immunodeficiency Virus infection (19, 33). On a molecular level, it has been shown that during infection, inflammatory markers like interleukin-6 (IL-6) prolong ventricular myocyte action potential by inhibiting the hERG potassium channel in cardiac myocytes, providing a direct mechanistic link between QTc prolongation and inflammation (34). Our findings are in line with the findings from the study by Gulletta et al. (7) where elevated CRP levels during hospital admission in individuals with COVID-19 were correlated with a prolonged QTc interval. Additionally, Rubin et al. (6) demonstrated that elevations of IL-6 were associated with QTc interval prolongation. However, no adjusted analysis was performed in either study to assess the relationship between QTc interval and levels of inflammatory markers, which is especially important as QTc interval prolongation is usually multifactorial and hospitalized patients can have multiple independent risk factors for QTc interval prolongation. Our study adds to the existing literature linking inflammation and ventricular repolarization in the largest sample of individuals with COVID-19 infection to date by showing an interval change in the QTc interval, irrespective of confounding factors in an adjusted analysis. Furthermore, we show a significant interval change in QTc with high vs. low CRP values independent of the type of QTc interval correction method used. Despite the significant interval increase in the median QTc value (15 ms with higher CRP values), the median QTc value remained in the normal range. Given the skewed QTc distribution to higher values, a disproportionate QTc prolongation >500 ms was observed in multiple patients (Figure 2).

### Limitations

There are several limitations worth noting. First, while our study is the largest cohort of patients evaluating the association between acute COVID-19 infection and QTc interval, the overall sample size remains modest. Second, while the retrospective nature of this study limits its generalizability, all individuals in this study served as their own controls, thus decreasing the risk of bias due to confounders. Third, to support the general effects of inflammation on QTc interval, reporting trends of QTc interval in association with inflammatory markers other than CRP would be helpful, however, the very small sample sizes for serial measurements of additional inflammatory markers (IL-6, ferritin) precluded a meaningful statistical analysis with these markers in our study. Prior studies have found the prolongation of the QTc interval with high IL-6 and ferritin levels, (6, 14) however, it is currently unknown which inflammatory marker correlates best with metrics of ventricular repolarization. Fourth, we could not adjust for specific QTc prolonging medications, or their doses given small numbers, however, the number of QTc prolonging medications was not statistically different when CRP values were high vs. low. Fifth, we could not adjust for other possible confounding variables such as potassium and magnesium levels, creatinine level, PR interval, or QRS interval as our goal was to minimize the number of variables included in the adjusted model due to the overall modest sample size. However, we have adjusted for variables that were associated with exposure or outcome in our sample. Finally, our study only included samples with marked elevations of CRP levels, as previous literature suggested a relationship between inflammatory markers and QTc interval with marked changes. Larger sample size studies are required in the future to explore a dose-dependent relationship between changes in CRP levels and the QTc interval.

## Conclusion

In summary, significant elevations of the inflammatory marker CRP are independently associated with prolongation of the QTc interval in hospitalized COVID-19 patients. Markedly elevated CRP may contribute to interval QTc prolongation, which may significantly increase arrhythmic risk. These findings may help guide clinicians when considering potential QTc prolonging medications or increased monitoring for arrhythmias in the setting of COVID-19.

## Data Availability Statement

The data analyzed in this study is subject to the following licenses/restrictions. The JH-CROWN registry is a collection of information about patients having suspected or confirmed COVID-19 infection. Access to the dataset can be requested upon Institutional Review Board approval. Requests to access these datasets should be directed to ictr@jhmi.edu.

## Ethics Statement

The studies involving human participants were reviewed and approved by the Johns Hopkins Institutional Review Board (IRB00248707). Written informed consent for participation was not required for this study in accordance with the national legislation and the institutional requirements.

## Author Contributions

AB and NI: conception and design. AB, NI, ME, DB, RM, LY, BM, AH, TM, and HC: analysis and interpretation of data, drafting of the manuscript or revising it critically for important intellectual content, and final approval of the manuscript submitted. All authors contributed to the article and approved the submitted version.

## Funding

This study was supported by a research grant from the Lovin' Every Day Foundation, Milton, Georgia.

## Conflict of Interest

Relationship with industry: AB has received consulting fees from Sanofi, Sarepta and AltaThera. The remaining authors declare that the research was conducted in the absence of any commercial or financial relationships that could be construed as a potential conflict of interest.

## Publisher's Note

All claims expressed in this article are solely those of the authors and do not necessarily represent those of their affiliated organizations, or those of the publisher, the editors and the reviewers. Any product that may be evaluated in this article, or claim that may be made by its manufacturer, is not guaranteed or endorsed by the publisher.

## Abbreviations

COVID-19: Coronavirus disease 2019
CRP: C-reactive protein
IL-6: Interleukin-6
QTc: corrected QT.
