# Supplementary material for: C-reactive Protein Elevation Is Associated With QTc Interval Prolongation in Patients Hospitalized With COVID-19
Source: Front Cardiovasc Med. 2022 Jun 23;9:866146. doi: 10.3389/fcvm.2022.866146 (PMC9261932; doi:10.3389/fcvm.2022.866146)
Supplement: Supplementary file 1 [file Data_Sheet_1.pdf]

Online Supplement

eTable 1. Crude association between QTc interval and potentially confounding variables using Mixed Effects Linear Regression.

	**Crude estimates**
**Variable**	β coefficient (95% CI)
**Female Sex**	15 (3.21 – 27)
**Race**	
**White vs Black**	4.76 (-9.64, 19.15)
**Other vs Black**	-11.73 (-24.83 – 1.35)
**BMI, kg/m^2**	0.005 (-0.75 – 0.8)
**WHO score**	2.92 (-0.8 – 6.64)
**HR, bpm**	0.15 (-0.13 – 0.43)
**PR Interval, ms**	-0.07 (-0.54 – 0.55)
**QRS duration, ms**	0.01 (-0.58 – 0.53)
**Number of QTc-prolonging medications**	
**1 vs 0**	16.34 (3.38 – 29.3)
**≥2 vs 0**	8.31 (-7.97 – 24.6)
**Creatinine, mg/dL**	1.38 (-2.08 – 4.83)
**Potassium, mmol/L**	3.75 (-7.2 – 14.66)
Abbreviations: bpm = beats per minute, BMI = body mass index, CRP = C reactive protein, HR= heart rate, ms=milliseconds, QTc = corrected QT interval, WHO = World Health Organization	

eFigure 1. Quantiles of each variable reported in Table 1 plotted against quantiles of normal distribution

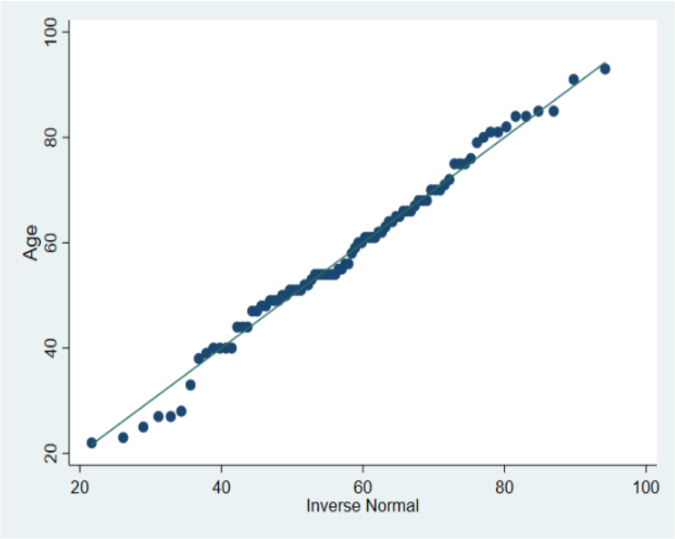

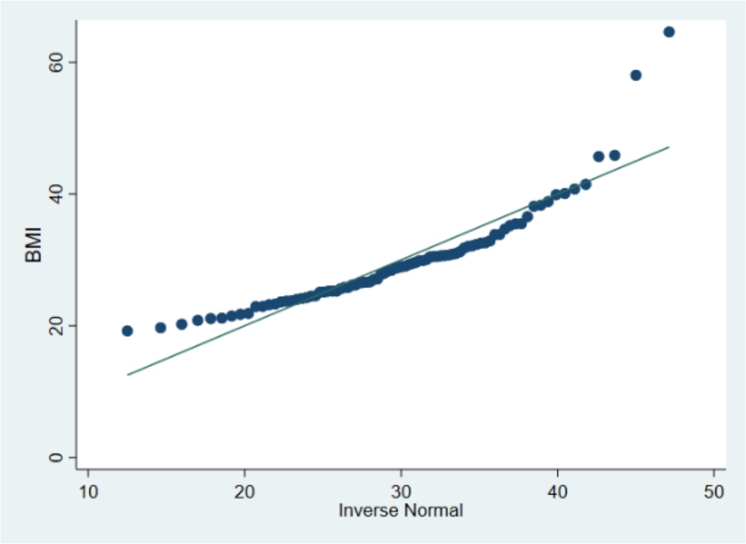

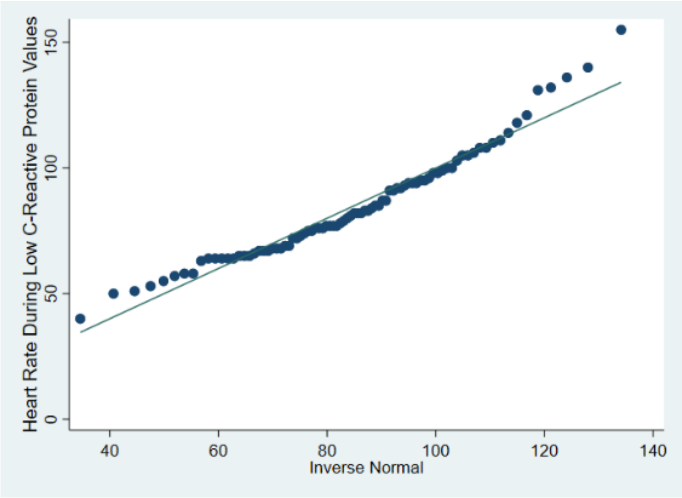
Heart Rate During Low C-Reactive Protein Values
0
50
100
150
40
60
80
100
120
140
Inverse Normal

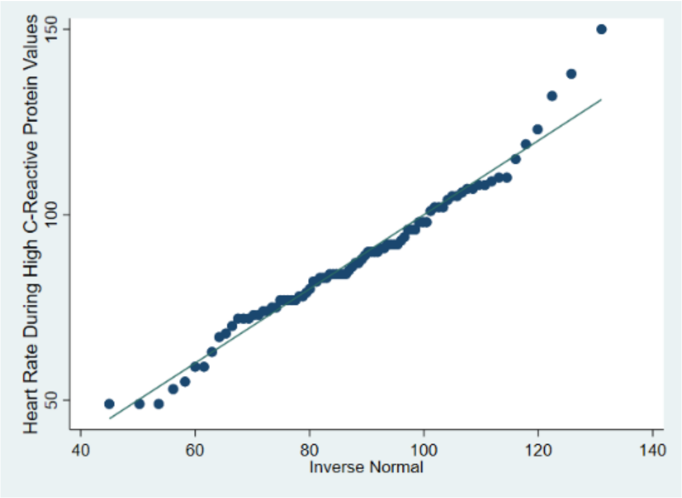
Heart Rate During High C-Reactive Protein Values
50
100
150
40
60
80
100
120
140
Inverse Normal

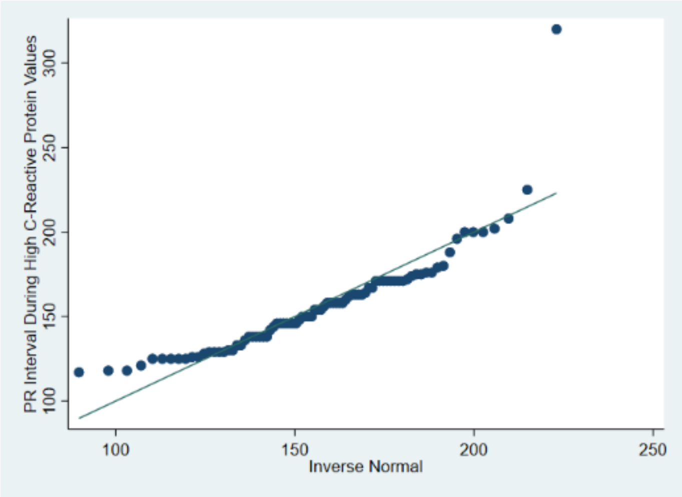
PR Interval During High C-Reactive Protein Values
100
150
200
250
300
100
150
200
250
Inverse Normal

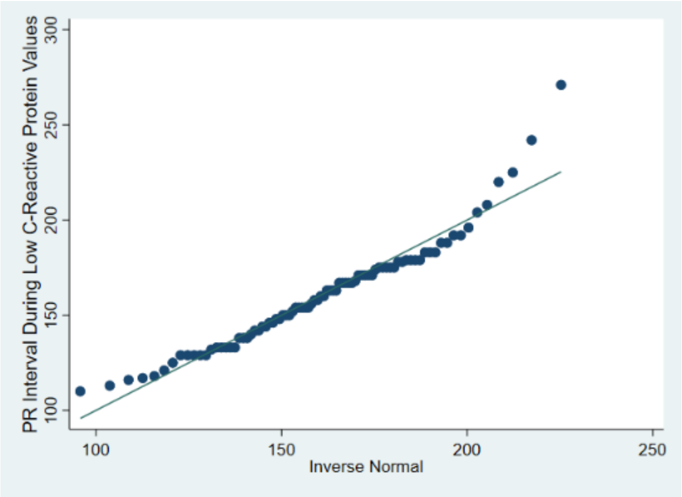
PR Interval During Low C-Reactive Protein Values
100
150
200
250
300
100
150
200
250
Inverse Normal

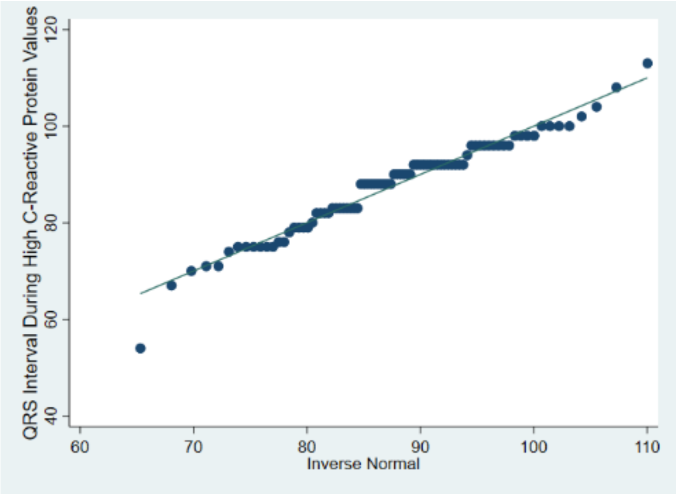
QRS Interval During High C-Reactive Protein Values
40
60
80
100
120
60
70
80
90
100
110
Inverse Normal

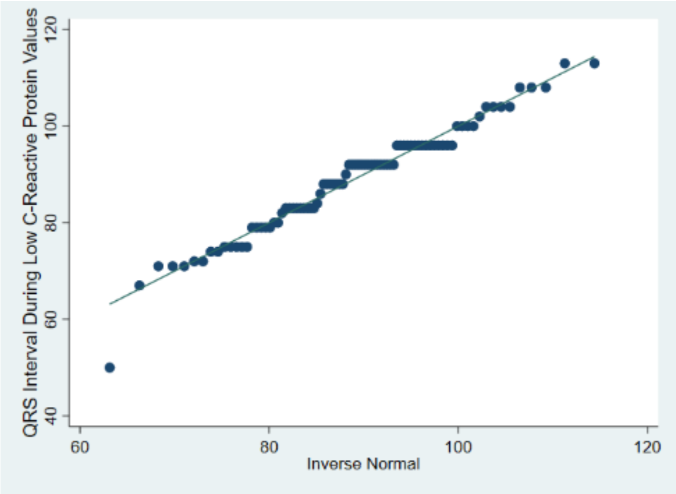
QRS Interval During Low C-Reactive Protein Values
40
60
80
100
120
60
80
100
120
Inverse Normal

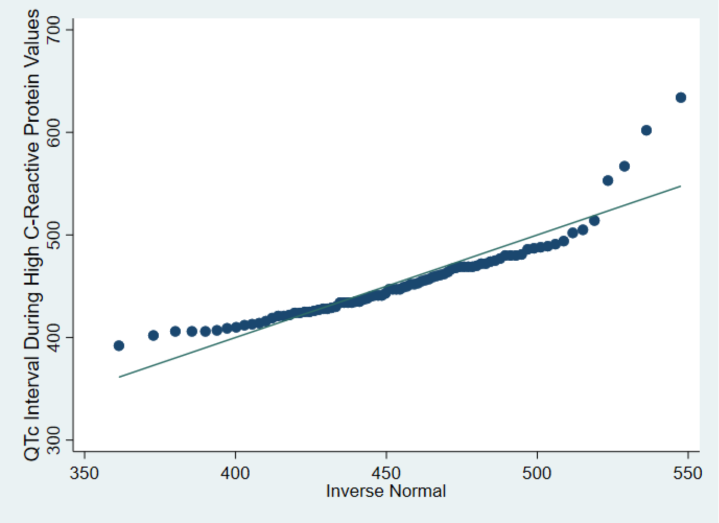
QTc Interval During High C-Reactive Protein Values
300
400
500
600
700
350
400
450
500
550
Inverse Normal

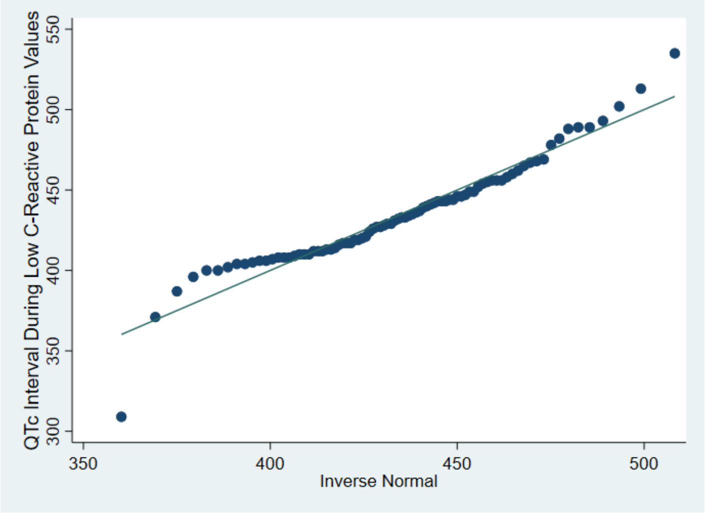
QTc Interval During Low C-Reactive Protein Values
300
350
400
450
500
550
350
400
450
500
Inverse Normal

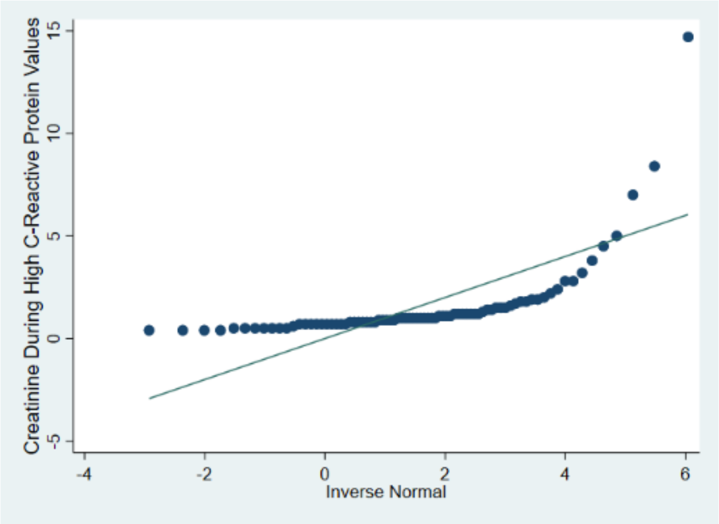
Creatinine During High C-Reactive Protein Values
-5
0
5
10
15
-4
-2
0
2
4
6
Inverse Normal

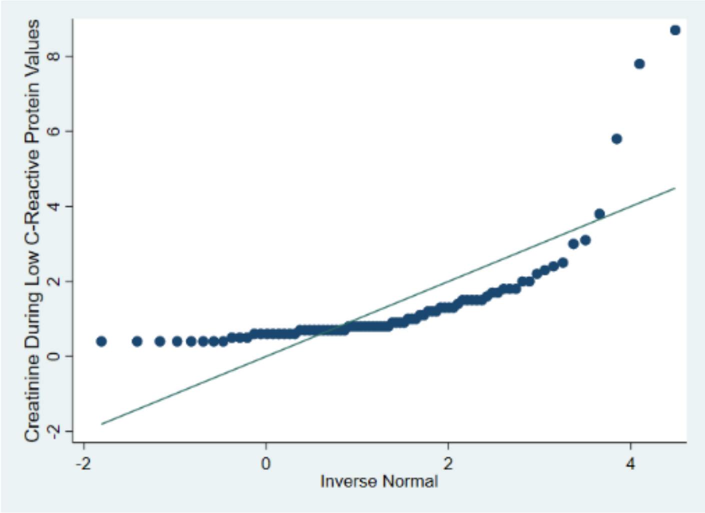
Creatinine During Low C-Reactive Protein Values
-2
0
2
4
6
8
-2
0
2
4
Inverse Normal

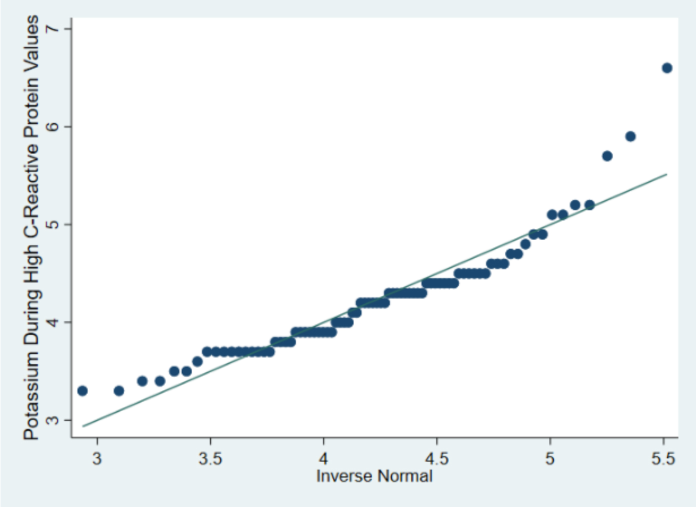
Potassium During High C-Reactive Protein Values
3
4
5
6
7
3
3.5
4
4.5
5
5.5
Inverse Normal

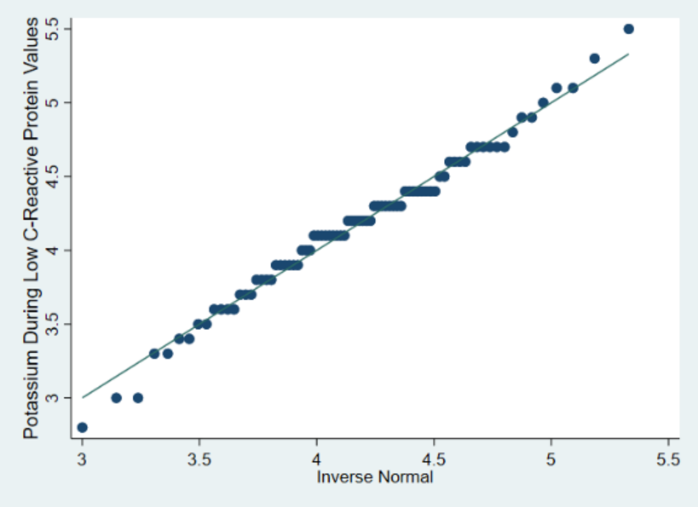
Potassium During Low C-Reactive Protein Values
3
3.5
4
4.5
5
5.5
3
3.5
4
4.5
5
5.5
Inverse Normal

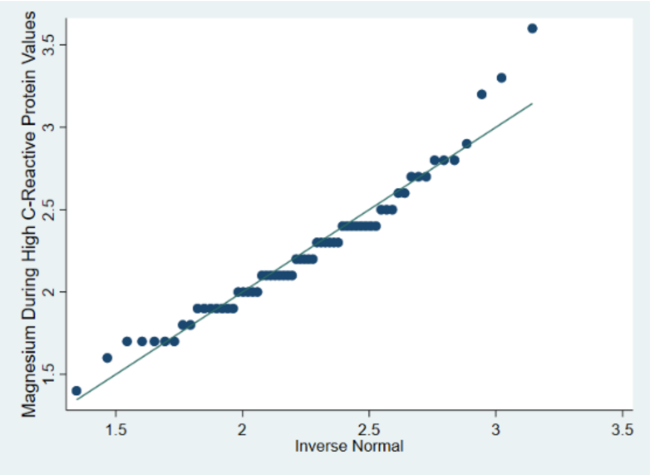

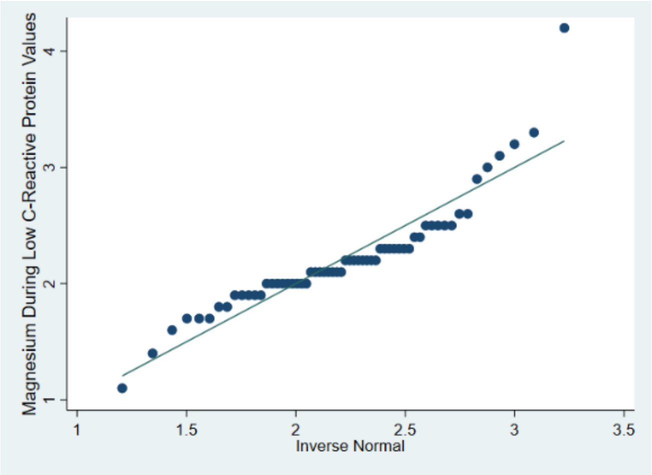

BMI = Body Mass Index

eFigure 2

Hospitalized adult patients admitted to five hospitals in Maryland and Washington, DC, area with Coronavirus disease 2019 infection **(n=2,456)**

Eligibility criteria:

Serial and paired measurements of electrocardiogram (ECG) and C-reactive protein on two sequential dates during the same hospitalization, one defined as "high" when CRP was five or more times above the upper limit of normal and the other defined as "low" when CRP level was less than fifty percent of the peak value. QRS duration of < 120 milliseconds (ms) and no evidence of atrial fibrillation on ECG.

Study Sample **(n=85)**
